# The association between anserine bursa pain and fall susceptibility: a prospective analysis of the osteoarthritis initiative

**DOI:** 10.3389/fragi.2025.1666627

**Published:** 2025-11-19

**Authors:** Fugui Wang, Jianhua Xiao, Zhi Song, Chao Zhang, Zhengyuan Zhu

**Affiliations:** 1 Department of Orthopedics, Jiangxi Dexing People’s Hospital, Dexing, China; 2 Department of Critical Care Medicine, Jiangxi Dexing People’s Hospital, Dexing, China

**Keywords:** accidental fall, elderly, osteoarthritis, pain, knee joint

## Abstract

**Background:**

Anserine bursa pain (ABP) is hypothesized to correlate with early-stage knee osteoarthritis (KOA). This research seeks to investigate the link between ABP and the incidence of falls in patients diagnosed with KOA or those at heightened risk of developing this condition.

**Method:**

The study utilized 2-year follow-up data derived from the Osteoarthritis Initiative cohort, a comprehensive multicenter observational investigation. Assessment of ABP was conducted through a tenderness/pain evaluation of the anserine bursa, while fall occurrences were self-reported by participants. Binary logistic regression was conducted, adjusting for confounding variables, and results were expressed as ORs along with their 95% CIs.

**Results:**

Data from 3,654 participants were analyzed, with 33.3% reporting ABP. Throughout the follow-up duration, 1,668 participants recorded instances of falls, 1,986 reported no falls, and 244 experienced recurrent falls annually. The primary outcome focused on incident falls, revealing a significant association between ABP and the odds of incident falls over the 2-year follow-up (odds ratio (OR) = 1.188, 95% Confidence interval (CI): 1.021–1.383, P = 0.026). When recurrent falls were included as an additional outcome, ABP was found to markedly predict the odds of these incidents over the 2-year follow-up (OR = 1.422, 95% CI: 1.065–1.898, P = 0.017). Sensitivity analyses confirmed the robustness of these findings among female participants and those without a prior history of falls.

**Conclusion:**

Within individuals diagnosed with KOA or those at substantial risk for KOA, a significant association exists between ABP and increased odds of falling over the 2-year follow-up.

## 1 Introduction

Falls represent a significant contributor to morbidity and mortality among the elderly demographic. Statistics provided by the World Health Organization indicate that between 28% and 35% of individuals aged 65 and older experience falls annually, with this rate escalating to between 32% and 42% for those aged 70 and above (Mercy et al., 2024). The repercussions of falls are profound, leading to severe physical injuries such as fractures and traumatic brain injuries, which can result in lasting functional disabilities and increased mortality (Zou et al., 2023; Mekkodathil et al., 2020). A study focusing on elderly patients hospitalized due to fall-related incidents found that approximately 36% sustained head or spinal injuries, typically contingent on the fall’s height and environmental context (Mekkodathil et al., 2020; Zhang et al., 2021). Furthermore, falls can adversely affect the mental wellbeing of seniors, as many develop a persistent fear of subsequent falls, restricting their daily activities and potentially leading to social isolation and diminished quality of life (Yu et al., 2020; Curl et al., 2020). Consequently, the early identification of elderly individuals at risk of falls is critical for enhancing their overall health and alleviating the strain on healthcare systems (Callaghan et al., 2015).

Numerous risk factors contribute to falls, encompassing biological, environmental, and socioeconomic dimensions (Zhao et al., 2024; Alabdullgader and Rabbani, 2021; Zhang et al., 2018). Pain, including its interference and distribution, has emerged as an independent predictor of injurious falls (Cai et al., 2021). lower-limb pain is closely linked to declines in balance and physical functionality among the elderly, thereby increasing the odds of falling over the 2-year follow-up (consistent with the study’s follow-up duration) (Hirase et al., 2020). Pain not only hampers physical activity but can also instill a fear of falling, further constraining engagement in daily activities (Castaldi et al., 2022; Mat et al., 2020). Prior investigations have indicated that knee symptoms, rather than radiographic osteoarthritis (ROA), are more strongly associated with increased odds of falling, typically evaluated through overall knee pain assessments (Cai et al., 2022). Nonetheless, pain localized to the anserine bursa area, which incorporates multiple tendons and ligaments, remains underexplored in relation to its impact on fall susceptibility. Inadequate stability of the lower limbs can lead to an increased likelihood of falls. Notably, individuals diagnosed with knee osteoarthritis (KOA) or those who are at a heightened risk of developing this condition may be particularly susceptible to anserine bursa pain (ABP). It has been estimated that roughly 20% of patients with OA also experience pes anserine bursitis, a condition that is especially prevalent among the elderly population (Uysal et al., 2015). Furthermore, the potential presence of muscle weakness, balance deficiencies, and various comorbidities associated with knee OA may elevate the risk of falls in these patients (Manlapaz et al., 2019). Consequently, it is essential to investigate the association between ABP and the incidence of falls within this vulnerable demographic.

Currently, there is a scarcity of research studies exploring the association between ABP and falls. In light of this background, the present study seeks to elucidate the relationship between the baseline presence of ABP and the subsequent incidence of falls among individuals with knee OA or those at risk for its development. We hypothesize that those suffering from ABP will demonstrate a higher propensity for falls in comparison to those without ABP. The findings of this study are intended to equip clinical practitioners with insights regarding the physical health and disease progression of patients with knee OA and ABP who are classified as high-risk.

## 2 Methods

### 2.1 Database and participants

The Osteoarthritis Initiative (OAI) is a multicenter observational cohort study dedicated to knee osteoarthritis. This study recruited 4,796 male and female participants aged between 45 and 79 years, either exhibiting symptomatic knee osteoarthritis or at risk for developing the condition (For example, obesity, age, gender, etc.). Participants were sourced from four clinical sites: Brown University in Rhode Island, The Ohio State University in Columbus, the joint facility of the University of Maryland/Johns Hopkins University in Maryland (with two distinct clinic locations), and the University of Pittsburgh (https://nda.nih.gov/oai/). Exclusion criteria encompassed inflammatory arthritis, significant narrowing of both knee joint spaces, unilateral knee replacement, severe constriction of the contralateral knee joint space, and reliance on walking aids. Data for this longitudinal cohort study were gathered at baseline and during subsequent evaluations, with a lack of ABP data for the second year, thus establishing a follow-up period of 2 years. The OAI cohort study received approval from the institutional review boards of the participating facilities, and all participants provided written informed consent to partake in this study. Therefore, our hospital has exempted ethical approval for this study. This study was conducted in accordance with the Declaration of Helsinki, which outlines the ethical principles for medical research involving human subjects, ensuring the protection of participants’ rights, safety, and wellbeing. All human participants in this study voluntarily provided informed consent to participate. They were fully informed of the study’s purpose, procedures, potential risks, and benefits, and explicitly expressed their willingness to participate in the research project.

**FIGURE 1:**
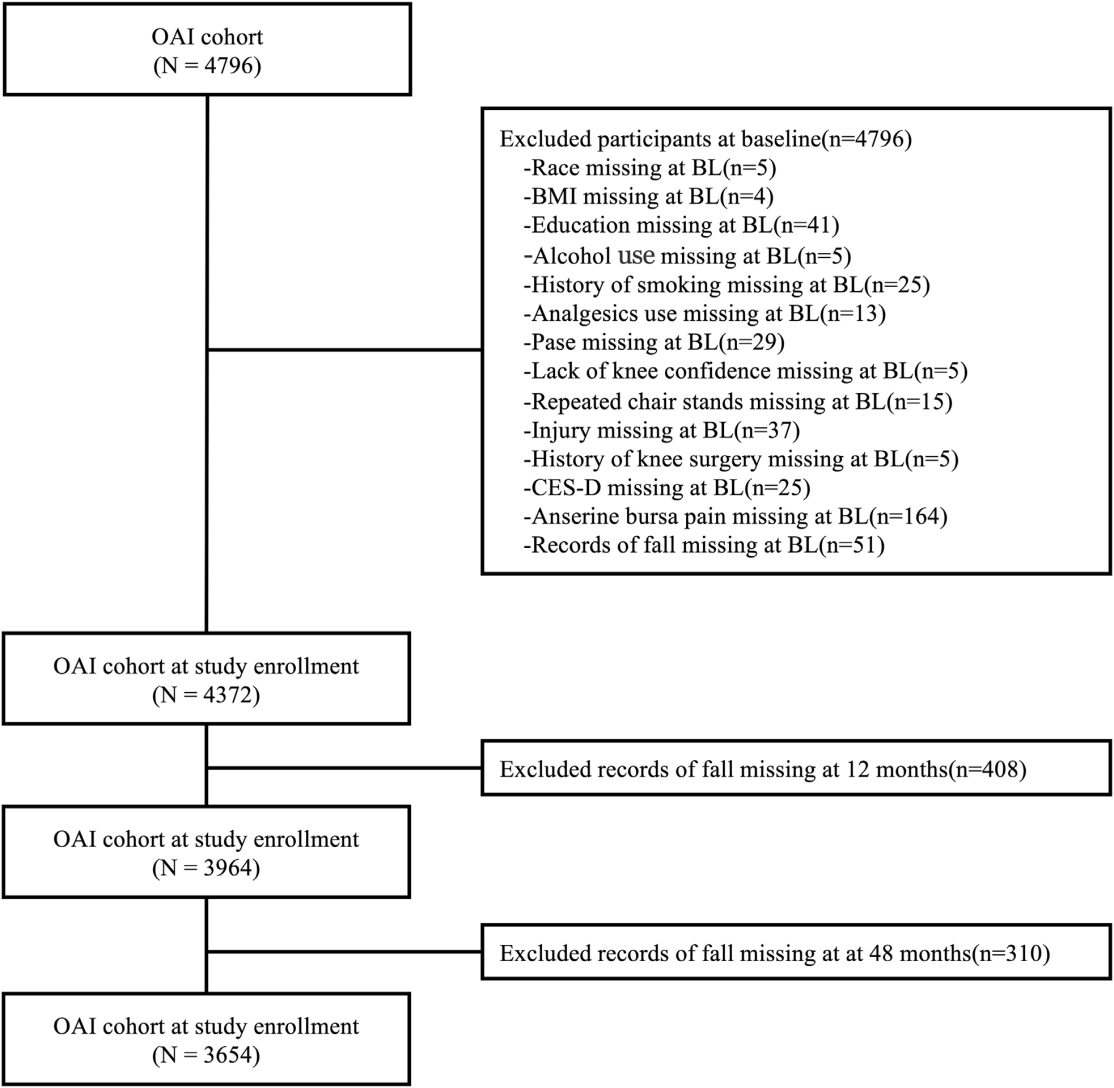
Flow chart of selection criteria.

Participants who were missing data regarding race, body mass index (BMI), educational attainment, alcohol consumption history, smoking history, analgesic usage history, Physical Activity Scale for Older Adults (PASE) score (Washburn et al., 1993), knee confidence, repeated chair stands (Shea et al., 2018), history of knee injury and surgery, Center for Epidemiological Studies Depression (CES-D) score (Cosco et al., 2020), and history of falls were excluded. Ultimately, a total of 3,654 participants were analyzed to investigate the association between ABP and incident falls (Figure 1). We divided participants into two groups based on the presence or absence of ABP and conducted a prospective analysis in the existing cohort. Notably, the excluded subjects shared similar demographic characteristics with those selected for the study.

### 2.2 Independent variable: ABP

Healthcare professionals underwent thorough centralized training prior to conducting knee examinations, which were carried out under the supervision of physicians at each site. Clinical specialists executed pain and tenderness assessments on the anserine bursa located beneath the medial aspect of the joint line, 2 cm from the patellar tuberosity (Skills in Rheumatology et al., 2021). Personnel responsible for clinical examinations participated in the center’s training and performed knee evaluations under expert supervision at each site. Comprehensive operational manuals for knee examinations are accessible online (https://nda.nih.gov/oai/study_documentation.html). Due to the possibility that palpation pressure may alter the reproducibility of these observations, examiners typically use a Chatillon dolorimeter to calibrate manual pressure. Previous studies have established that the assessment results of ABP have high reliability across different locations (Cibere et al., 2004).

### 2.3 Dependent variable: incidence of falls

Falls are defined as “Fallen and landed on floor or ground in the past 12 months” (Deandrea et al., 2010). Additionally, participants provided self-reported data regarding the frequency of falls they experienced over the past year, with responses categorized as 0, 1, 2, 3, 4, 5, or 6 or more falls. The outcomes were evaluated at baseline and during the 12- and 24-month follow-up intervals. At the conclusion of each data collection phase, participants were asked to report the number of falls they had encountered in the preceding year by responding to the inquiry: “Did you fall in the past year?” In this investigation, individuals who reported experiencing two or more falls annually were classified as recurrent fallers.

### 2.4 Covariate

The following variables were assessed at baseline visit: age, BMI, gender, race, education, history of smoking, alcohol use, analgesics use, history of fall, PASE, repeated chair stands, knee confidence, history of knee injury, history of knee surgery, and CES-D. Race was categorized as Caucasian or non-Caucasian, BMI as either normal or obese, education as postgraduate or non-postgraduate, and smoking status as current versus never-smokers. The repeated chair stands assessment required participants to complete a minimum of five repetitions. The knee confidence questionnaire, derived from the Knee Injury Osteoarthritis Outcome Score, asked respondents, “How much are you troubled with a lack of confidence in your knees?” (Sharma et al., 2015). A history of knee injury was defined as an incident that severely impaired the participant’s walking capacity for at least 1 week.

### 2.5 Statistical analysis

Participants were classified into two groups based on whether they experienced a fall during the follow-up period. Data pertaining to continuous variables conformed to a normal distribution, as verified by the Kolmogorov-Smirnov test. The baseline characteristics of the participants were presented as means with standard deviations (SD) or as counts and percentages. p-values were determined utilizing Fisher’s Exact test for frequency comparisons, alongside independent t-tests for mean assessments. A Binary Logistic regression analysis was executed, with the occurrence of falls during the follow-up period designated as the outcome variable and the presence of ABP at baseline identified as the primary exposure (as a binary variable). Two models were formulated: an unadjusted model and a multivariate model. Stratified analysis was conducted to discern the influence of ABP on fall prediction across diverse populations. The strength of association between baseline variables and corresponding outcomes was expressed in terms of odds ratios (OR) and their 95% confidence intervals (CIs). All statistical evaluations were carried out using SPSS software (version 27.0; IBM SPSS Inc., Armonk, New York, United States of America), with statistical significance established at p < 0.05.

## 3 Result

### 3.1 Participant characteristics

**FIGURE 2:**
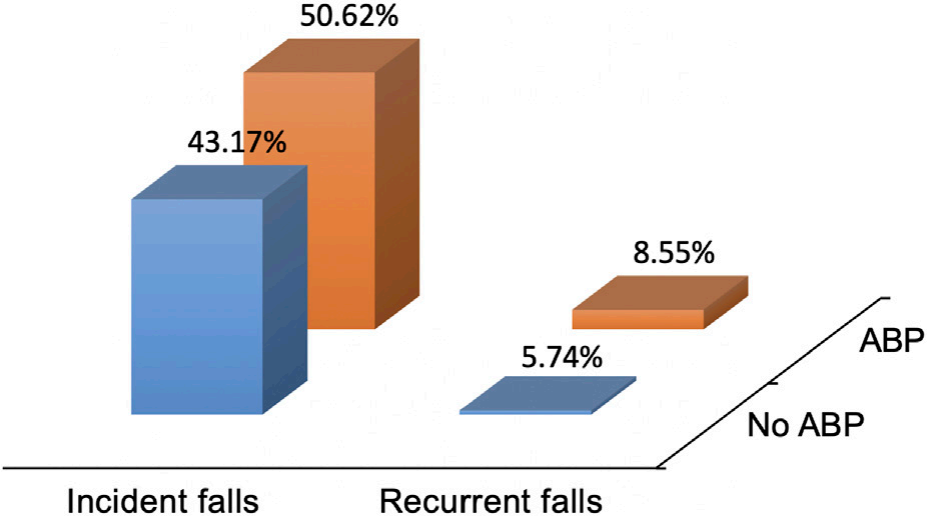
The incidence of falls and recurrent falls during baseline follow-up of goose foot sac pain status. Incident falls are falls assessed during follow-up and refer to falls that have occurred since baseline assessment. Recurrent falls refer to falls that occur at least twice a year during the follow-up period.

Table 1 shows the combined data of demographic characteristics and research variables. Overall, ABP at baseline affected 33.3% of participants, with 1668 individuals experiencing incident falls, and 244 having frequent falls. Compared to participants who reported no falls during the follow-up period, those who experienced incident falls were more likely to be female (p < 0.0001), but no age differences were observed (p = 0.74). The BMI reported by those who experienced incident falls was higher (p = 0.0043), as was medication use (p = 0.0006), while there were no significant differences in race (p = 0.07), post-college education (p = 0.06), drinking history (p = 0.59), smoking history (p = 0.16), PASE (p = 0.32), repeated chair-standing test (p = 0.61), history of knee injury (p = 0.16), and history of knee surgery (p = 0.08). Additionally, they were more depressed (p < 0.0001), and reported a higher incidence of previous falls at baseline (p < 0.0001), as well as a significantly higher prevalence of ABP at baseline (p < 0.0001). Furthermore, as shown in the data of Figure 2, participants without ABP reported a lower incidence of falls (43.17%), and a lower incidence of recurrent falls each year (5.74%).

**TABLE 1:** The demographic characteristics of the study.

Characteristic	No incident falls (n = 1986)	Incident falls (n = 1668)	*P*-value
Age, mean (SD)	61.4 (9.1)	61.5 (9.1)	0.74
Female, n (%)	1075 (54.1)	1050 (62.9)	**<0.0001**
White, n (%)	1618 (81.5)	1397 (83.8)	0.07
BMI (kg/m^2^), mean (SD) Post college education, n (%) Alcohol use, n (%) History of smoking, n (%) Analgesics use, n (%) PASE (m/s), mean (SD) Lack of knee confidence, n (%) Repeated chair stands, n (%)	28.36 (4.54) 773 (38.9) 1615 (81.3) 899 (45.3) 437 (22) 160.67 (80.21) 1025 (51.6) 1900 (95.7)	28.81 (4.96) 700 (42) 1368 (82) 794 (47.6) 448 (26.9) 163.36 (82.11) 923 (55.3) 1590 (95.3)	**0.0043** 0.06 0.59 0.16 **0.0006** 0.32 **0.0246** 0.61
Injury, n (%)	815 (41)	723 (43.3)	0.16
Surgery, n (%) CES-D, mean (SD) Previous falls, n (%)	454 (22.9) 5.74 (6.06) 389 (19.6)	341 (20.4) 6.91 (7.26) 797 (47.8)	0.08 **<0.0001** **<0.0001**
Positive anserine bursa, pain/tenderness, n (%)	601 (30.3)	616 (36.9)	**<0.0001**

BMI: body mass index; SD: standard deviation; PASE: physical activity scale for the elderly score; Repeated chair stands: able to complete 5 stands; CES-D: center for epidemiologic studies depression score. Bold indicates that p is less than 0.05, which is statistically significant.

### 3.2 The association between ABP and incident falls over 24 months

Table 2 elucidates the relationship between the presence of ABP at baseline and incident falls within a 24-month period, examined both in the overall population and within stratified subgroups. The analysis of incident falls as an outcome indicates that ABP participants are more likely to experience incident falls than those without ABP ([OR]: 1.349, 95% confidence interval [CI]: 1.175–1.549, p < 0.001). Following the adjustment for baseline variables such as age, BMI, gender, race, education level, smoking status, alcohol consumption, analgesic use, prior fall history, PASE, repeated chair stands, knee confidence, prior knee injury, knee surgery history, and CES-D scores, the association between baseline ABP and incident falls within 24 months remained statistically significant (OR: 1.188, 95% CI: 1.021–1.383, p = 0.026). When stratifying by baseline fall history, among individuals with no prior fall history, participants with ABP exhibited a 1.412-fold higher odds of incident falls over the 2-year follow-up compared to those without ABP (95% CI: 1.186–1.682, P < 0.001). Even after controlling for confounding factors, those with ABP maintained a 1.258-fold higher odds of incident falls over the 2-year follow-up relative to their non-ABP counterparts (95% CI: 1.046–1.513, P = 0.015). Furthermore, when stratified by gender, age, and CES-D scores, patients with ABP younger than 65 years and with CES-D scores below 16 demonstrated a 1.489-fold (95% CI: 1.243–1.782, P < 0.001) and a 1.377-fold (95% CI: 1.190–1.592, P < 0.001) higher odds of incident falls over the 2-year follow-up, respectively, compared to participants without ABP. However, among those aged 65 years and older at baseline, as well as those with CES-D scores of 16 or higher, no significant association was identified between ABP and incident falls. After adjusting for confounding variables, the risk of incident falls for ABP patients younger than 65 years and with CES-D scores below 16 remained 1.275 times (95% CI: 1.046–1.554, P = 0.016) and 1.221 times (95% CI: 1.041–1.432, P = 0.014) higher, respectively, compared to non-ABP participants. Moreover, among female patients with ABP, the adjusted analysis indicated an increased likelihood of incident falls (OR: 1.244, 95% CI: 1.034–1.498, p = 0.021). However, there was no significant association established between ABP and incident falls among participants who were aged 65 or older at baseline, had CES-D scores of 16 or higher, or were male, even after accounting for confounding variables.

**TABLE 2:** Association of ABP with incidence of falls over 24 months.

Characteristic	Unadjusted model		Multivariate model	
	OR (95% CI)	*P*	OR (95% CI)	*P*
All^a^	1.349 (1.175, 1.549)	**<0.001**	1.188 (1.021, 1.383)	**0.026**
No history of falls^b^	1.412 (1.186, 1.682)	**<0.001**	1.258 (1.046, 1.513)	**0.015**
History of falls^b^	1.118 (0.868, 1.441)	0.388	1.029 (0.787, 1.346)	0.834
Female^c^	1.244 (1.047, 1.479)	**0.013**	1.244 (1.034, 1.498)	**0.021**
Male^c^	1.290 (1.009, 1.650)	**0.042**	1.096 (0.837, 1.435)	0.506
Age<65^d^	1.489 (1.243, 1.782)	**<0.001**	1.275 (1.046, 1.554)	**0.016**
Age≥65^d^	1.173 (0.945, 1.455)	0.148	1.077 (0.848, 1.368)	0.543
CES-D<16^e^	1.377 (1.190, 1.592)	**<0.001**	1.221 (1.041, 1.432)	**0.014**
CES-D≥16^e^	0.958 (0.614, 1.496)	0.852	1.000 (0.601, 1.665)	0.999

^a^Multivariate Model: Adjusted by age, body mass index (BMI), gender, race, education, history of smoking, alcohol use, analgesics use, history of fall, PASE, repeated chair stands, knee confidence, history of knee injury, history of knee sugury and Center for Epidemiologic Studies Depression (CES-D). ^b^Multivariate Model: ^a^Multivariate Model without history of fall. ^c^Multivariate Model: ^a^Multivariate Model without gender. ^d^Multivariate Model: ^a^Multivariate Model without age. ^e^Multivariate Model: ^a^Multivariate Model without CES-D. Bold indicates that p is less than 0.05, which is statistically significant.

### 3.3 Association between ABP and recurrent falls over 24 months

In Table 3, the analysis results with recurrent fall events as the endpoint show that ABP participants had higher odds of recurrent falls over the 2-year follow-up than those without ABP (OR: 1.533, 95% CI: 1.178–1.996, p < 0.001). After adjusting for confounding factors, ABP at baseline was significantly associated with higher odds of recurrent falls over the 2-year follow-up (OR: 1.422, 95% CI: 1.065–1.898, p = 0.017). Participants were stratified according to whether they had a fall history at baseline, and among those without a baseline fall history, the odds of recurrent falls over the 2-year follow-up for participants with ABP was 2.013-fold that of participants without ABP (95% CI: 1.172–3.457, P = 0.011). After adjusting for confounding factors, the odds of recurrent falls over the 2-year follow-up for participants with ABP was still 1.810-fold higher than that of participants without ABP (95% CI: 1.021–3.211, P = 0.042). However, there was no significant association between ABP and recurrent falls among participants with a fall history at baseline. Additionally, stratified by gender, age, and CES-D, the risk of recurrent falls for female and ABP patients with CES-D < 16 was 1.611 times (95% CI: 1.145–2.266, P = 0.006) and 1.555 times (95% CI: 1.172–2.064, P = 0.002) higher, respectively, than for participants without ABP. However, among participants with age ≥65 years and CES-D ≥ 16 at baseline, there was no significant association between ABP and recurrent falls. After adjusting for confounding factors, the risk of recurrent falls for female and ABP patients with CES-D < 16 was still 1.558 times (95% CI: 1.079–2.248, P = 0.018) and 1.489 times (95% CI: 1.090–2.034, P = 0.012) higher than for participants without ABP. Interestingly, after adjusting for confounding factors, ABP patients with age ≥65 years had a higher risk of recurrent falls (OR: 1.880, 95% CI: 1.142–3.034, p = 0.013). However, after adjusting for confounding factors, there was no significant association between ABP and recurrent falls among participants with age <65 years, CES-D ≥ 16, and males.

**TABLE 3:** Association of ABP with recurrent falls over 24 months.

Characteristic	Unadjusted model		Multivariate model	
	OR (95% CI)	P	OR (95% CI)	P
All^a^	1.533 (1.178, 1.996)	0.001	1.422 (1.065, 1.898)	0.017
No history of falls^b^	2.013 (1.172, 3.457)	0.011	1.810 (1.021, 3.211)	0.042
History of falls^b^	1.280 (0.932, 1.757)	0.127	1.317 (0.941, 1.842)	0.109
Female^c^	1.611 (1.145, 2.266)	0.006	1.558 (1.079, 2.248)	0.018
Male^c^	1.492 (0.953, 2.337)	0.080	1.210 (0.738, 1.984)	0.449
Age<65^d^	1.495 (1.078, 2.074)	0.016	1.269 (0.885, 1.821)	0.195
Age≥65^d^	1.683 (1.074, 2.640)	0.023	1.880 (1.142, 3.095)	0.013
CES-D<16^e^	1.555 (1.172, 2.064)	0.002	1.489 (1.090, 2.034)	0.012
CES-D≥16^e^	1.208 (0.581, 2.513)	0.613	1.027 (0.454, 2.323)	0.949

^a^Multivariate Model: Adjusted by age, body mass index (BMI), gender, race, education, history of smoking, alcohol use, analgesics use, history of fall, PASE, repeated chair stands, knee confidence, history of knee injury, history of knee sugury and Center for Epidemiologic Studies Depression (CES-D). ^b^Multivariate Model: ^a^Multivariate Model without history of fall. ^c^Multivariate Model: ^a^Multivariate Model without gender. ^d^Multivariate Model: ^a^Multivariate Model without age. ^e^Multivariate Model: aMultivariate Model without CES-D. Bold indicates that p is less than 0.05, which is statistically significant.

## 4 Discussion

In this investigation, we identified that anserine bursa pain (ABP) serves as a significant predictive variable for the odds of both initial falls and recurrent falls over the 2-year follow-up among individuals with osteoarthritis or those predisposed to developing the condition. Our results demonstrate that the relationship between ABP and falls remains consistent even after accounting for potential confounding variables, including age, body mass index (BMI), sex, and symptoms of depression.

Prior research has recognized knee pain as a crucial element linked to an elevated risk of falls. A study focusing on the elderly population revealed a notable association between knee pain and fall incidents. Specifically, the existence of knee pain heightened the odds of falls in older adults by 54% (Smith et al., 2018). Moreover, another investigation indicated that the intensity of knee pain is positively related to the frequency of falls, implying that greater pain severity correlates with increased odds of falling (Hu et al., 2021). ABP and medial knee pain due to osteoarthritis need to be distinguished. Medial knee osteoarthritis is characterized by tenderness in the medial joint space, often accompanied by crepitus (Nigoro et al., 2021; Sekiya et al., 2022; Duffaut et al., 2023). It may result in limited knee range of motion and morning stiffness. Pain in the pes anserinus is characterized by tenderness 2 cm below the medial side of the knee, and the compression test is positive. It generally does not present with joint space tenderness or crepitus (Xiong et al., 2023; Nur et al., 2018; Johns et al., 2021). Importantly, in individuals without a prior history of falls, the odds for both new and recurrent falls associated with ABP over the 2-year follow-up is significantly amplified, suggesting that ABP may act as an early indicator of fall vulnerability. A longitudinal study indicated that even among participants who had not experienced any falls in the preceding year, their self-assessment of health status was substantially linked to the likelihood of future falls (Porto et al., 2020). Additionally, another study indicated that inadequate lower limb strength could be a critical risk factor contributing to the first fall (Porto et al., 2021). Hannington et al. found that in patients experiencing knee pain, both the location and intensity of the pain significantly influenced their load tolerance during unilateral knee extension, suggesting that knee pain may compromise their lower limb strength (Hannington et al., 2022). Patients with a history of falls exhibit heightened levels of concern and alertness regarding fall risks. However, these individuals frequently endure an increased fear of falling, which may prompt them to engage in avoidance behaviors in their daily routines, consequently impacting their quality of life and functional abilities (Bhorade et al., 2021). Elderly individuals who have previously fallen often refrain from participating in activities that could result in further falls. While this behavior may stem from self-preservation instincts, it can inadvertently lead to a decline in physical capabilities (Kader et al., 2016).

The influence of ABP on fall occurrences appears to differ among various age groups. Specifically, for individuals younger than 65 years, ABP demonstrates efficacy in forecasting the odds of incident falls over the 2-year follow-up, whereas for those aged 65 years and above, the association between ABP and the odds of recurrent falls over the 2-year follow-up is more pronounced. It is important to highlight that reports of single falls may be attributed to chance (Hoffman et al., 2018; Lo-Ciganic et al., 2017). Fall risk awareness tends to decline with advancing age (Moreira et al., 2018). Younger participants, who are more cognizant of their fall risk, tend to perform better on functional assessments and participate in a greater number of physical activities. This heightened awareness may explain the absence of a significant association between ABP and recurrent falls in the under-65 age group in this investigation. Younger individuals might possess a greater awareness of their fall risk compared to their older counterparts, thus decreasing their chances of experiencing multiple falls. Furthermore, gender and depressive states are crucial factors influencing the association between knee pain and fall risk. The findings of the study indicate that women frequently report higher levels of psychological stress and dysfunction associated with knee pain, which in turn elevates their risk of falling (Abdeen, 2020). Additionally, the link between ABP and falls is significant among individuals without depression, but not among those who are depressed. Nevertheless, depression may also heighten fall risk in older adults, and these psychological factors could further influence their awareness of falls and coping mechanisms (Cui et al., 2023). Consequently, in research examining falls as a primary outcome, the effect of ABP on falls in depressed patients may be obscured by the direct influence of depression itself.

ABP frequently coexists with muscle weakness, diminished confidence in the knee, and compromised proprioception, which can result in altered gait patterns and compensatory strategies aimed at alleviating pain, ultimately increasing the odds of falling over the 2-year follow-up. Moreover, knee discomfort may deter individuals from engaging in movement, leading to a sustained state of immobility (e.g., full knee extension). As a result, patients may be inclined to avoid bearing weight on the medial aspect of the knee, causing abnormal gait patterns which can disrupt the alignment and load distribution across the hip, knee, and ankle. This malalignment of body weight can lead to a decline in functional ability, a change that may become apparent as early as 18 months into monitoring (Sharma et al., 2001). The pain below the inner side of the knee caused by ABP increases the risk of falls. Therefore, it is recommended to incorporate ABP assessment into the fall risk system, supplementing two specific physical examination signs: tenderness at the pes anserinus and the squeeze test. Additionally, an assessment of pain-related functional limitations should be included, such as single-leg standing duration and time taken to go up and down stairs, to quantify functional impact. A positive result in these two indicators can serve as early warning factors for fall risk, avoiding missed judgments of “pain-mediated high-risk populations” based solely on traditional indicators. For interventions targeting this population, the first step is to control the source of pain. During the acute phase, ice application can help reduce inflammation; if symptoms persist, ultrasound-guided injection of anti-inflammatory medication may be considered. After pain relief, isometric contraction training for the quadriceps and other muscles surrounding the knee should be implemented to strengthen the muscle power. This should be combined with balance training and gait correction to address gait deviations and restore movement stability. In daily life, patients should be guided to avoid actions that exacerbate the pull on the pes anserinus, such as squatting, kneeling, or climbing stairs, and to choose soft-soled shoes to reduce the risk of falls during pain episodes. However, utilizing short-term nonsteroidal anti-inflammatory drugs, enhancing proprioception, strengthening muscles, and optimizing gait may can mitigate fall risk. For instance, interventions such as physical therapy and functional rehabilitation training are recognized as effective approaches to improving balance and gait among the elderly, thereby assisting patients in regaining confidence and decreasing fall incidents (Oh-Park et al., 2019; Ikpeze et al., 2015).

In spite of the merits associated with this investigation, certain limitations must be acknowledged. This research employs a retrospective cohort design, which inherently raises the potential for recall bias stemming from its dependence on self-reported information. Additionally, the exclusive focus on the Osteoarthritis Initiative database may restrict the applicability of the findings to broader populations. Furthermore, while our analysis accounted for numerous confounding factors, the influence of unmeasured variables, such as the presence of additional chronic conditions, cannot be disregarded as they may skew the outcomes. Future inquiries should aim to assess interventions targeting ABP with the intent to alleviate the risk of falls. This study did not consider the temporal changes in ABP pain status and therefore cannot reflect the long-term association between the dynamic evolution of ABP pain and fall risk. Future research should analyze the long-term longitudinal effects of ABP on fall risk to further enhance the research conclusions. A comprehensive approach that integrates gait training, muscle-strengthening regimens, and psychological support is essential to tackle the complex nature of fall risk linked to ABP. Gaining insight into the interplay between ABP, mobility, and psychosocial elements is vital for mitigating fall incidents and enhancing the quality of life for individuals with knee osteoarthritis or those predisposed to the condition.

## 5 Conclusion

In conclusion, the findings of this investigation demonstrate that ABP serves as a predictive marker for the odds of incident and recurring falls over the 2-year follow-up in individuals diagnosed with knee osteoarthritis or those identified as being at elevated odds of developing this condition, especially among patients who have not experienced falls previously and within the female demographic. Alongside the current literature on ABP, the outcomes of this research advocate for the implementation of fall prevention strategies targeted at ABP patients and the importance of early intervention, which may contribute to a decrease in fall rates within this vulnerable group and enhance their overall quality of life.

## Data Availability

Publicly available datasets were analyzed in this study. This data can be found here: https://nda.nih.gov/oai/.

## Abbreviations

ABP, Anserine bursa pain; KOA, knee osteoarthritis; ORs, Odds ratios; Cis, Confidence intervals.
